# Author Correction: Experimental evidence on improving COVID-19 vaccine outreach among migrant communities on social media

**DOI:** 10.1038/s41598-022-26500-8

**Published:** 2022-12-20

**Authors:** Jasper Tjaden, Esther Haarmann, Nicolai Savaskan

**Affiliations:** 1 Department of Economic and Social Sciences, University of Potsdam, Potsdam, Germany; 2 International Organization for Migration, Global Migration Data Analysis Centre, Berlin, Germany; 3 Department of Public Health Neukölln, District Office Neukölln of Berlin, Blaschkoallee 32, 12359 Berlin, Germany

Correction to: *Scientific Reports*, 10.1038/s41598-022-20340-2, published on 28 September 2022

The original version of this Article contained an error in Affiliation 3, which was incorrectly given as ‘District of Neukoelln, Government of Berlin, Berlin, Germany’.

The correct affiliation is listed below.

Department of Public Health Neukölln, District Office Neukölln of Berlin, Blaschkoallee 32, 12359, Berlin, Germany

The original Article has been corrected.

